# A Probe Into the Psychological Mechanism of Generation Z College Students Using Network Buzzwords in the Internet Era

**DOI:** 10.3389/fpsyg.2021.663728

**Published:** 2021-08-02

**Authors:** Dandan Dong

**Affiliations:** School of Journalism and Communication, Nanjing University, Nanjing, China

**Keywords:** educational psychology, college students, linguistics, network buzzwords, psychology mechanism

## Abstract

Network buzzwords are ubiquitous in the internet era. To explore the psychology of college students using network buzzwords, the development trend and characteristics of network buzzwords are analyzed first. A questionnaire survey is performed to learn how college students use network buzzwords currently; then, the obtained data are analyzed and discussed. Results demonstrate that 81.59% of college students can understand what the buzzwords mean. As for psychological factors, the average score of the identity dimension is the highest, and the average score of social support is the lowest. Among the behavioral factors, the communication factor scores the highest. Regarding the overall psychological and behavioral factors, in general, boys score higher than girls, and science and engineering students score higher than those majoring in literature, history, and art. Students of higher grades score lower than those of lower grades regarding using network buzzwords in communication and entertainment activities. The psychological mechanism of Generation Z college students using network buzzwords has been explored, which can provide scientific and practical reference materials for subsequent analysis and research on online behaviors of college students and is also of great significance for internet education of college students.

## Introduction

The accelerated development of the internet has brought significant impacts and changes to many aspects of human society. Internet technology has an incredibly massive impact on the daily lives of people. Tremendous changes have occurred in the daily lives of people in the internet era (Ma’azi and Janfeshan, 2018; Kojima et al., 2019). According to statistics, by the end of 2019, the number of internet users in China exceeded 850 million, and the internet penetration rate exceeded 60% (Koenraadt and van de Ven, 2018). Generation Z refers to the group born between 1995 and 2009 (Seemiller and Grace, 2017; Chunta et al., 2021). The academic community believes that Generation Z spends more time and energy on the internet than other populations, while the internet exerts huge influences on college students. With the advent of the internet 4.0 era, the daily learning of college students has become closely connected with the internet (Prakash Yadav and Rai, 2017). As internet technology advances quickly, the lives of Generation Z are utterly different from those of other generations. This group has formed its unique lifestyle and communication styles. All-day online is a distinctive feature of Generation Z, and the online behavior of college students has become an all-day behavior (Mohr and Mohr, 2017). The influence of internet culture on Generation Z has increased, and the internet has become an indispensable necessity in their daily lives. Thus, Generation Z is the first group to accept and respond to internet culture and network buzzwords (Seemiller and Grace, 2017). Scholars have analyzed the connotation of network buzzwords from linguistics and researched these words from pedagogy, psychology, and sociology (Cluley, 2013; Chen et al., 2016; Penkler et al., 2019).

Rinker et al. (2016) stated that college students aged from 18 to 29 years are the dominant group of Chinese Internet users and that internet technology has a very important impact on the daily learning and life of college students (Rinker et al., 2016). Hennessy et al. (2019) found that the internet had become an important channel for college students to communicate with each other, express feelings, vent emotions, and voice opinions (Hennessy et al., 2019). Network buzzwords are very trendy among college students; they are one of the groups that use network buzzwords most frequently (Hong, 2016). However, due to the massive amount of information on the internet, the resulting network buzzwords are often two-sided. Some network buzzwords such as “positive energy” are positive and have good semantic connotations. The spread of positive network buzzwords can significantly improve ideological and moral levels of college students and construct a civilized and harmonious campus environment (Liu D. et al., 2017; Yuan, 2018). However, there are some uncivilized network buzzwords involving pornography, violence, and vulgarity. If these words spread, the ideological level of college students will be seriously affected, resulting in a negative impact on the construction of a harmonious campus and a civilized society (Luescher et al., 2017). At present, there are few research studies on the use of network buzzwords by college students, so it is of great theoretical value and practical significance to carry out such research studies. Therefore, in education management in colleges and universities, attention needs to be paid to the use of network buzzwords by college students, thereby correctly guiding the behaviors of college students (Bondar, 2018).

Literature analysis can comprehensively analyze the connotation of network buzzwords and the relationship between them and social development from a linguistic perspective. The current situation of college students using network buzzwords is the focus of educational psychology. In this study, questionnaire surveys and interviews are adopted. A scale to survey the situations of college students using network buzzwords is developed, and the surveyed data are analyzed statistically to discuss the psychological factors of college students. Based on the questionnaire survey results, psychological factors and behaviors of college students using network buzzwords can be understood, and the influences of gender, discipline, and age on college students using network buzzwords are explored and analyzed. This research presents reasonable contents, appropriate methods, and comprehensive results. The purpose of this study is to further explore network behavior habits of college students and provide a scientific and effective reference for subsequent research.

## Research Methods and Main Contents of the Questionnaire Survey

### Linguistic Analysis of Network Buzzwords

Network buzzwords are defined as a network language generated by the network and applied to the network at the same time. They are mainly used in network communication and have special meanings in specific media (Liu S. et al., 2017). From the perspective of linguistics, some researchers believe that network buzzwords are a special form of spoken language and are unique, lively, and fresh words that people use on the Internet. With the rapid development of network buzzwords, the content of network buzzwords has also undergone tremendous changes. Network buzzwords break through the language habits stipulated by written language and show their own distinctive characteristics (Shen et al., 2019).

From the perspective of language structure, network buzzwords mainly have the following characteristics. First, network buzzwords have a prominent nature of entertainment and are an important product of contemporary network culture and a significant reflection of netizens in real society. They mainly come from the spoof of netizens to express their own demands in the form of relaxed humor and alleviate the pressure on life and work (Lei, 2016). Second, network buzzwords are innovative. The innovation of network buzzwords is very obvious in terms of word formation and word meaning. There are relatively fewer restrictions and regulations in the cyber world than in the real world. Similarly, network buzzwords have also broken the constraints of relevant provisions of linguistics, so network buzzwords are clearly innovative in language composition. Third, network buzzwords are generally concise. They are usually short and succinct words, which obey “the economic principle of language” in linguistics (Kushalnagar et al., 2018). Finally, network buzzwords are easy to understand. It is essential to make network buzzwords easy to understand to ensure that most Internet users understand and accept them. Meanwhile, its understandability is mainly reflected in oral expression (Cao and Zhao, 2018). The understandability of network buzzwords also helps in realizing its application in different industries. Therefore, it is the understandability of network buzzwords that makes them favored by internet users of different occupations, educational backgrounds, and regions (Clothey et al., 2016).

Network buzzwords have experienced more than 10 years of evolution. At first, network buzzwords mainly had one single form, but with the development of communication, network buzzwords at this stage possess not only diverse forms but also rich ideological feelings. The emotions contained in online buzzwords of today can be roughly divided into positive emotions and negative emotions. Positive emotions mainly include praise, confidence, love, and expectation; while negative emotions mainly contain anger, surprise, ridicule, doubt, sadness, and disappointment (Pritzker, 2020).

### The Emotional and Psychological Mechanism of College Students Using Network Buzzwords

Emotions expressed by network buzzwords can be roughly divided into positive emotions and negative emotions (Wu et al., 2018; Sha and Jiugen, 2020). Positive emotions include praise, confidence, love, and expectation; while negative emotions include anger, surprise, ridicule, doubt, sadness, and disappointment. Network buzzwords with positive emotions can inspire the spirit of college students and promote their thinking. In contrast, negative emotions damage the thinking of students, causing depression and even desperation, which seriously affects the learning and lives of students (Weng, 2018; Xue and van Stekelenburg, 2018).

From the perspective of educational psychology, factors of college students using network buzzwords can be analyzed from four dimensions: motivation, social support, one’s own emotion, and identity. Motivation is the process by which people generate an internal driving force in using network buzzwords, which urges them to move toward goals expected by buzzwords (Lin et al., 2017). Social support refers to using some material or spiritual means to help people who use network buzzwords. One’s own emotion indicates various physical and psychological states while using network buzzwords (Doré et al., 2017). Identity refers to the attitude of the groups using network buzzwords (Larina et al., 2017). From the perspective of educational psychology, behaviors of college students using network buzzwords include getting information, communication, business trade, and leisure and entertainment. Getting information is the behavior of people to acquire original information from network buzzwords (Chow, 2016). Communication is the most common usage scenario of network buzzwords. Using network buzzwords in business trade can shorten the distance between buyers and sellers (Evans et al., 2017). To summarize, network buzzwords play a vital role in daily leisure and entertainment.

### Establishment of Questionnaire Survey Scale

**FIGURE 1:**
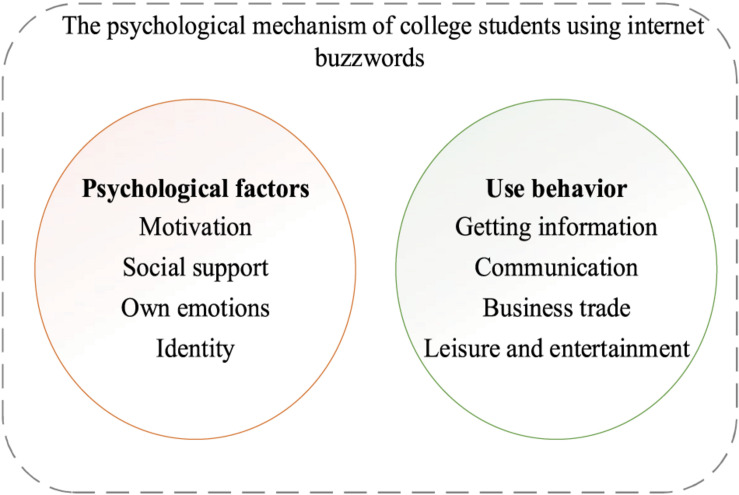
Specific performance of college students using network buzzwords.

According to the above results, the specific performance of college students using network buzzwords will be analyzed from two aspects, psychological factors and behavioral factors, in an effort to formulate the questionnaire based on this classification condition. The details are shown in Figure 1 below.

### Determination and Distribution of Survey Scales

Research methods are determined based on the basic concepts of network buzzwords, the psychological mechanism of college students using network buzzwords, and other reference materials. Questionnaire scales and interview outlines to survey the situations of college students using network buzzwords are constructed (Leavy, 2017).

The survey on college students using network buzzwords includes personal information of students, psychological analysis of college students using network buzzwords, and behavior analysis of college students using network buzzwords. Seven questions are set up to investigate the basic personal information of the students, namely, gender, age, grade, internet age, time spent on the internet every day, familiarity with network buzzwords, and attitudes toward network buzzwords. Psychological factors of college students using network buzzwords include motivation, social support, self-emotion, and sense of identity. Five questions are set for each factor. Behavioral factors of college students using network buzzwords include getting information, communication, business and trade, and leisure and entertainment, with five questions for each factor as well. The survey totals 45 questions, compiled using a five-point Likert scale. Options for each question are “very inconsistent,” “inconsistent,” “neither consistent nor inconsistent,” “consistent,” and “very consistent” (Chen, 2018; Hidayatulloh et al., 2019), corresponding to 1–5 points, respectively.

**FIGURE 2:**
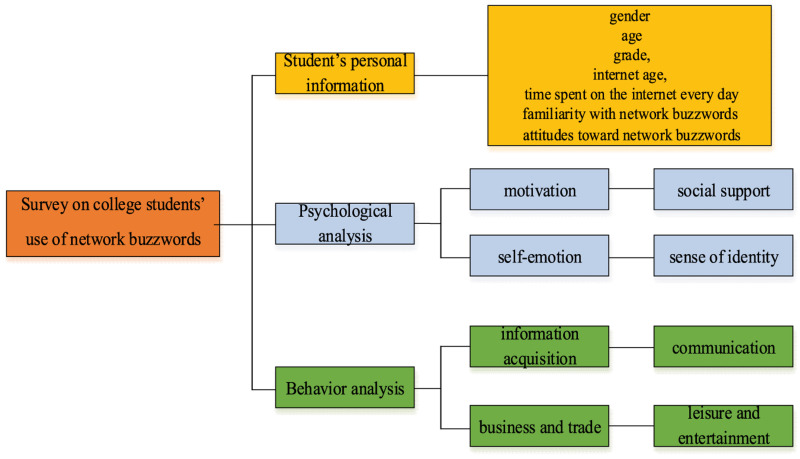
Overview of the overall questionnaire survey.

The specific content of the questionnaire survey is summarized in Figure 2.

### Determination of Interview Outline

The use of network buzzwords is associated with the actual situation of individuals. Hence, the interview method is adopted to explore the factors and current situation of college students using network buzzwords in detail. The interview is performed in the form of a symposium, and the topic is “the use of network buzzwords.” The interview includes three themes: opinions on network buzzwords, whether network buzzwords are frequently used, and the impact of network buzzwords on life. The research samples include students of all grades from the first to fourth year to make the interview cover as comprehensive a range as possible. The total interview time is about 2 h. This interview aimed to explore the use of network buzzwords among college students and provide more in-depth information for research.

### Basic Information of Research Samples

Questionnaire surveys are distributed offline and online. Research samples are college students from five universities in X City, S Province. These universities are of three different types: key universities, ordinary universities, and vocational-technical schools. A total of 400 questionnaires were distributed, and 388 were returned with a response rate of 97%. The basic information of research samples is summarized based on valid questionnaires, as shown in Table 1.

**TABLE 1:** Basic information on research samples.

Category	Content	Number of people
Gender	Male	183
	Female	205
Major	Literature and History	120
	Science and Engineering	187
	Art	81
Grade	First-grade	113
	Second-grade	102
	Third-grade	96
	Fourth-grade	75

Students of different grades, majors, and genders are interviewed, with the theme “the reason and connotation of using network buzzwords,” in an effort to enrich the empirical data and deeply understand the specific connotation of college students using network buzzwords. The basic situation of the interviewees is summarized in Table 2.

**TABLE 2:** Basic situation of interviewees.

Category	Content	Number of people
Gender	Male	26
	Female	30
Grade	First-grade	16
	Second-grade	13
	Third-grade	15
	Fourth-grade	12

According to Table 2, a total of 56 students are interviewed, including 30 girls and 26 boys; 16 first-grade students, 13 second-grade students, 15 third-grade students, and 12 fourth-grade students.

### Analysis Methods of the Reliability and Validity of the Questionnaire

The reliability and validity of the questionnaire are also investigated. The Cronbach’s α coefficient is used to measure the reliability of the scale. Generally, the value of Cronbach’s α coefficient more than 0.7 represents an effective reliability; and the closer to 1, the better was the reliability of the scale. Furthermore, the Kaiser–Meyer–Olkin (KMO) and Bartlett hemisphericity tests are adopted as the evaluation indexes in the validity analysis of the scale. The validity test required that the value of KMO be greater than 0.7. Besides, the IBM SPSS 25.0 software is used for statistics in reliability and validity analysis.

## Data Statistics and Analysis of the Questionnaire

### Analysis Results of the Reliability and Validity of the Questionnaire

**FIGURE 3:**
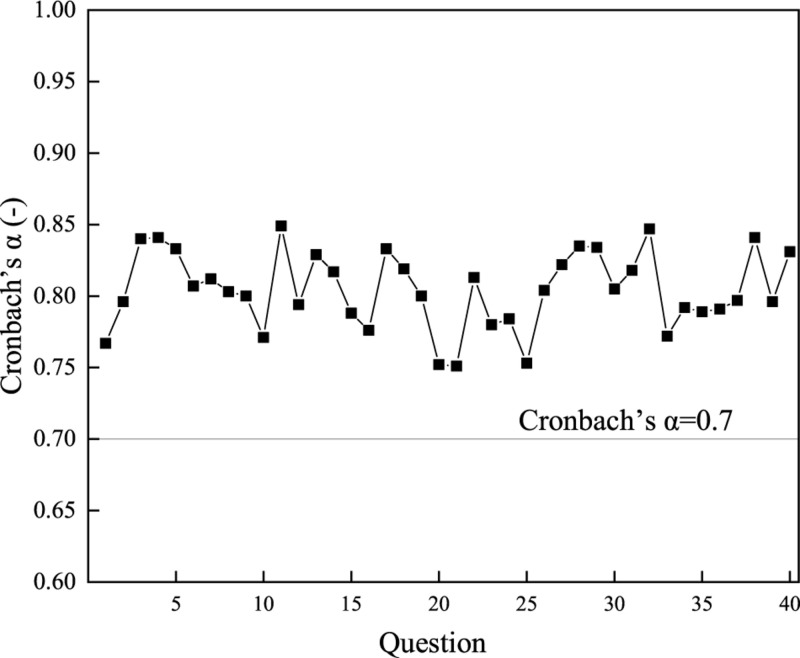
Analysis results of the reliability of the questionnaire.

Figure 3 and Table 3 illustrate the results of reliability and validity of the questionnaire, respectively.

**TABLE 3:** Analysis results of the validity of the questionnaire.

Type	Numerical value
KMO	0.715
Bartlett	675.578
Sig.	0.001

According to Figure 3, the Cronbach’s α coefficient of each question of the scale is more than 0.7, indicating that the scale has good reliability and can meet the requirements of the study. Table 3 shows that the KMO value of the scale is 0.715 (> 0.7), proving that the validity of the two scales is qualified. In summary, the scale used in the study has good reliability and validity and can be applied to practical research.

### Questionnaire Results of College Students Using Network Buzzwords

Network buzzwords have significant timeliness, changing very fast with time. Therefore, buzzwords that were popular in the past 2 years are selected as a survey indicator. First, the understanding of network buzzwords by college students is explored. A total of 20 network buzzwords are summarized from the second half of 2019 to the first half of 2020. Understanding more than 15 network buzzwords is regarded as “known very well”; understanding 10–14 network buzzwords is regarded as “a basic understanding”; understanding 5–9 network buzzwords is regarded as “do not know”; and understanding less than five network buzzwords is regarded as “know nothing.” The selected network buzzwords are summarized in Table 4.

**TABLE 4:** Selected network buzzwords.

Number	Network buzzwords	Number	Network buzzwords
1	Mutual learning between civilizations	2	Blockchain
3	Hardcore	4	Melting memes
5	There are thousands of roads, safety first	6	Green-eyed monster
7	996	8	It is too hard for me.
9	I do not care what you think; it is my opinions that matter.	10	Bullying
11	People are supreme, and life is supreme	12	Retrograde
13	Pretty cool	14	Waves
15	Mythical beast	16	Live-streaming sales
17	Double-loop	18	Worker
19	Involution	20	Humblebrag

**FIGURE 4:**
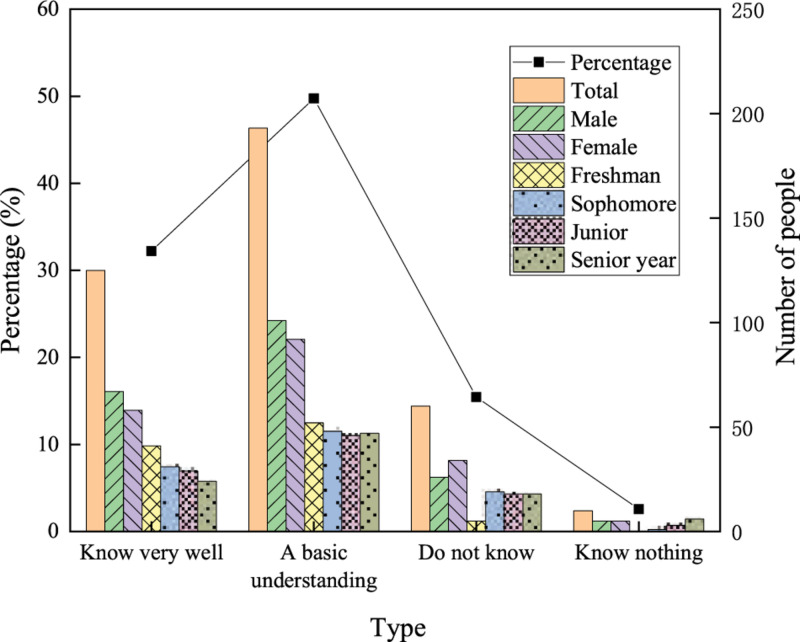
Understanding of network buzzwords of college students.

The degree of understanding of network buzzwords by college students is investigated, and the results are shown in Figure 4.

According to Figure 4, 125 out of the 388 college students know network buzzwords very well, accounting for 32.21%; 193 have a basic understanding of network buzzwords, accounting for 49.74%; 60 do not know network buzzwords, accounting for 15.46%; and only 10 students know nothing about network buzzwords, accounting for 2.57%. More boys choose “Know very well” and “A basic understanding” than girls, while more girls choose “Do not know” than boys. This result reveals that boys are more familiar with network buzzwords than girls. Regarding grades, more first-grade students choose “Know very well” and “A basic understanding” than students of other grades. Moreover, not a single first-grade student chooses “Know nothing,” proving that first-grade students use network buzzwords more frequently.

**FIGURE 5:**
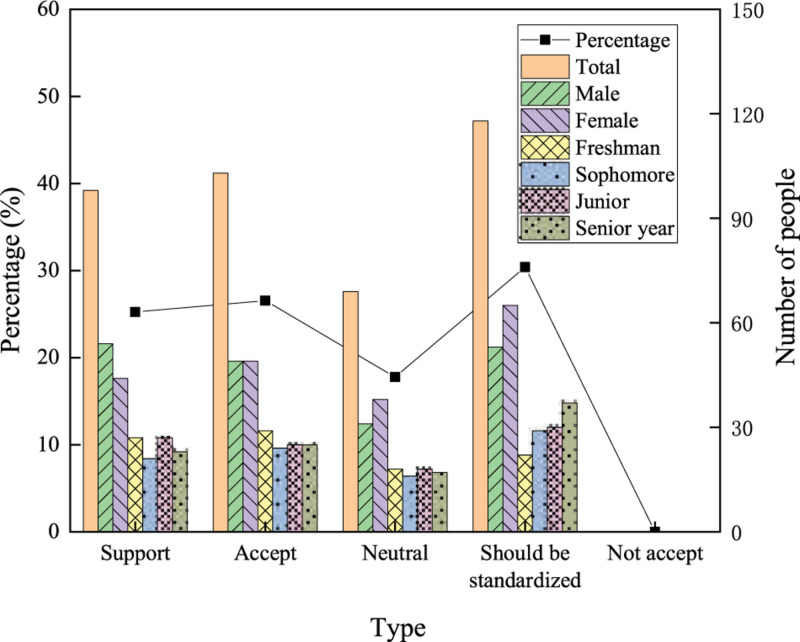
Acceptance of network buzzwords by the surveyed college students.

The attitudes of college students toward network buzzwords are explored. The options include “support,” “accept,” “neutral,” “should be standardized,” and “not accept.” The survey results are visualized in Figure 5.

Among the students surveyed, 98 support the spread and use of network buzzwords, accounting for 25.25%; 103 accept the use of network buzzwords, accounting for 26.54%; 69 hold a neutral attitude to network buzzwords, accounting for 17.78%; and 118 think that network buzzwords should be standardized, accounting for 30.4%. No one thinks that the use of network buzzwords should not be supported. There are no significant differences in the attitudes of boys and girls toward network buzzwords, and the male-to-female ratio in each option is not much different. Regarding age, more fourth-grade students choose “Should be standardized” than first-grade students.

The above two surveys reveal that college students are familiar with network buzzwords and that everyone holds a positive attitude toward network buzzwords. They accept and support the use of network buzzwords, but most of them think that the use of buzzwords should be standardized. Therefore, relevant departments need to regulate and manage network buzzwords to ensure the correct use of network buzzwords by college students and the healthy development of bodies and minds of students.

### Psychological Factors and Usage Behaviors of College Students Using Network Buzzwords

The maximum, minimum, and average values of the psychological factors of college students using network buzzwords are statistically analyzed.

**FIGURE 6:**
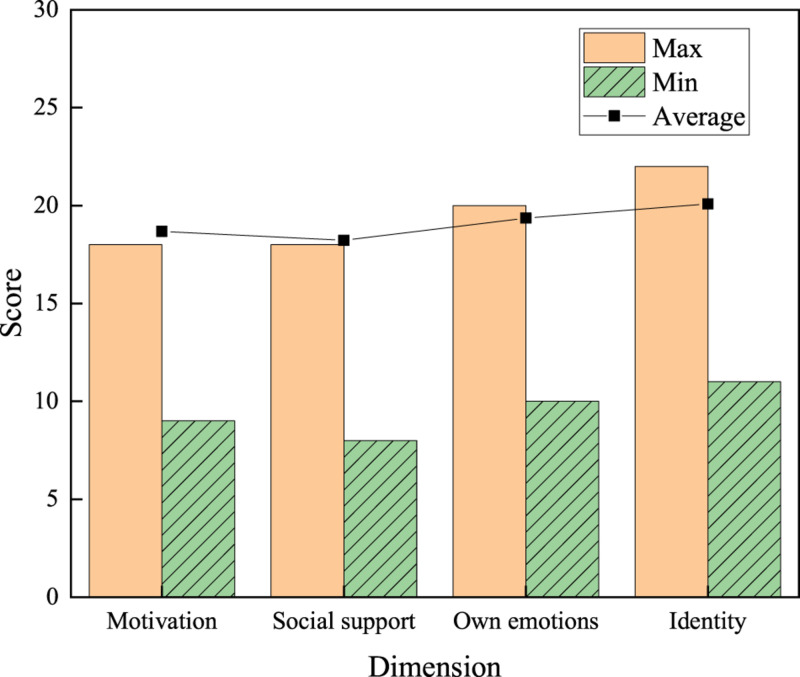
Scores of psychological factors.

Among the four psychological dimensions, the average score of the identity dimension is the highest, reaching 20.08 points. The average score of social support is 18.22 points. The scores of motivation and one’s own emotion are 18.68 and 20.08 points, respectively. Figure 6 shows that among the psychological factors of college students using network buzzwords, the sense of identity accounts for a larger proportion, while the social support accounts for a minor proportion.

The above results suggest that the sense of identity is critical among the psychological factors of college students using network buzzwords. In the daily communication and activities of college students, network buzzwords occupy a significant position. Using network buzzwords helps students to better integrate into the group and activate the chat atmosphere.

**FIGURE 7:**
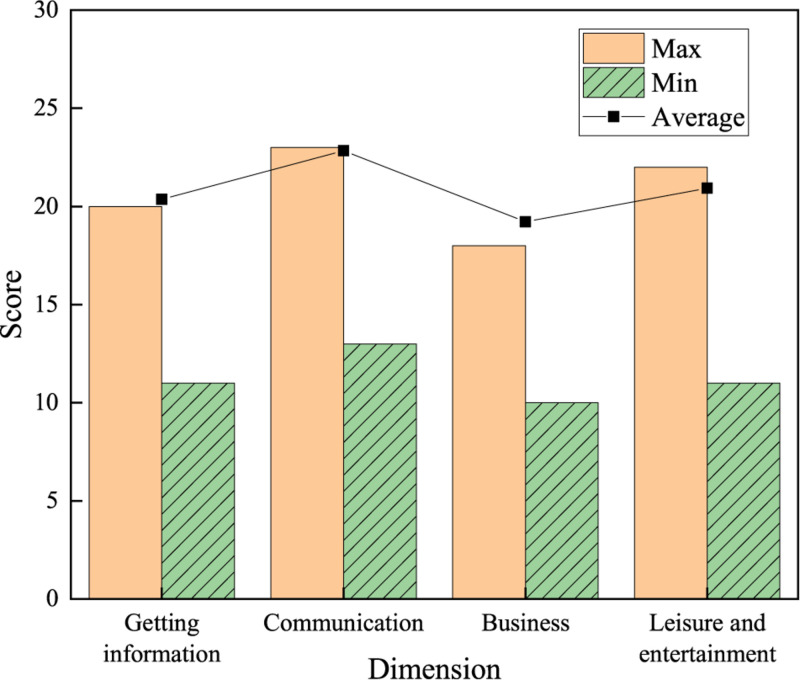
Scores of behavioral factors.

Results of the behavioral factors of college students using network buzzwords are shown in Figure 7.

Among the behavioral factors of college students using network buzzwords, the communication factor scores the highest, with an average score of 22.84 points, followed by leisure and entertainment, getting information, and business trade, with average scores of 20.93, 20.36, and 19.22 points, respectively. These results suggest that communication is the most critical behavior mode for college students to use network buzzwords.

### Factors Affecting College Students’ Use of Network Buzzwords

Influences of gender, majors, and grades on the use of network buzzwords by college students are surveyed and analyzed.

(1) Gender factor

**FIGURE 8:**
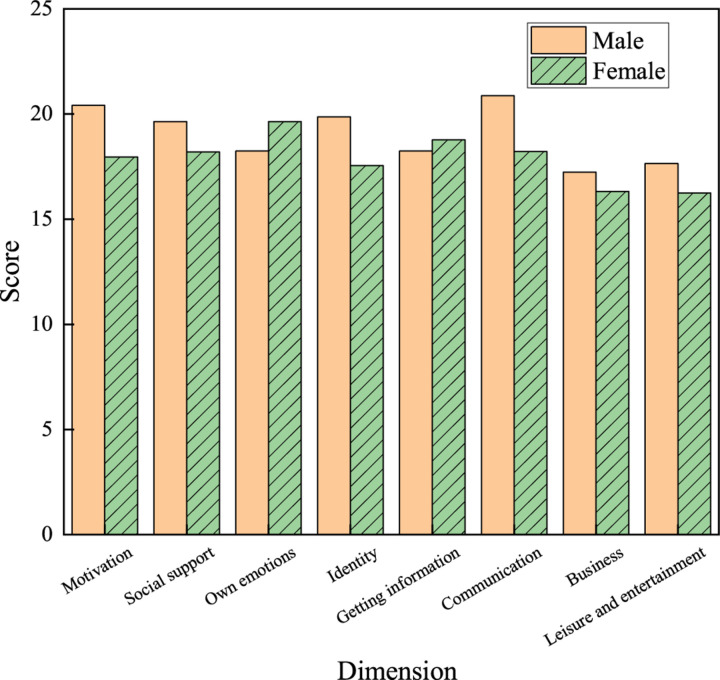
Influences of gender on the use of network buzzwords by college students.

Figure 8 shows the views of boys and girls on using network buzzwords. In terms of psychological factors, boys (18.24 points) only score lower than girls (19.64 points) in one’s own emotion dimension. In terms of behavioral factors, boys only score lower than girls in getting information dimension.

(2) Major factor

**FIGURE 9:**
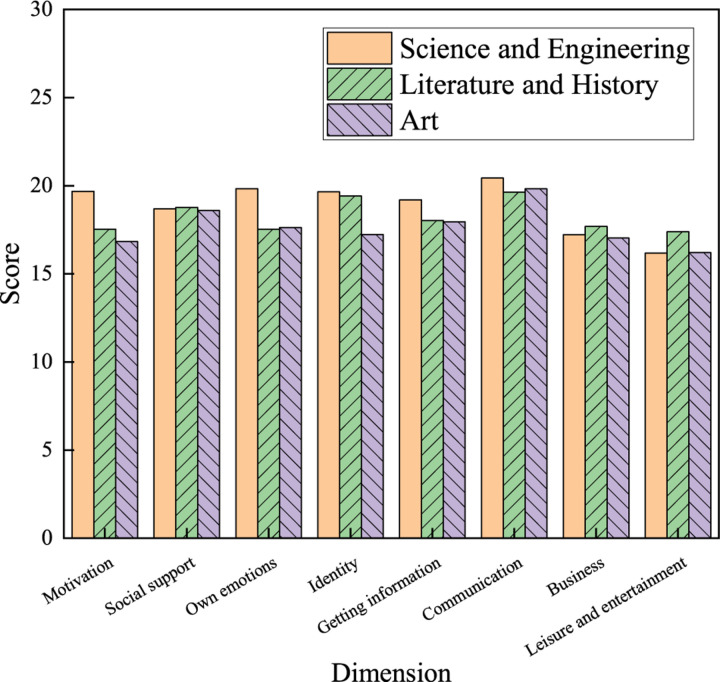
Influences of majors on the use of network buzzwords by college students.

As shown in Figure 9, students of different majors also have differences in using network buzzwords. Regarding psychological factors, the scores of college students majoring in science and engineering are generally higher than those majoring in literature, history, and art. Especially in motivation and one’s own emotional factors, the scores of science and engineering students are 19.68 and 19.84 points, which is significantly higher than those of other majors. Regarding behavioral factors, the differences between students of different majors are not very notable.

(3) Grade factor

**FIGURE 10:**
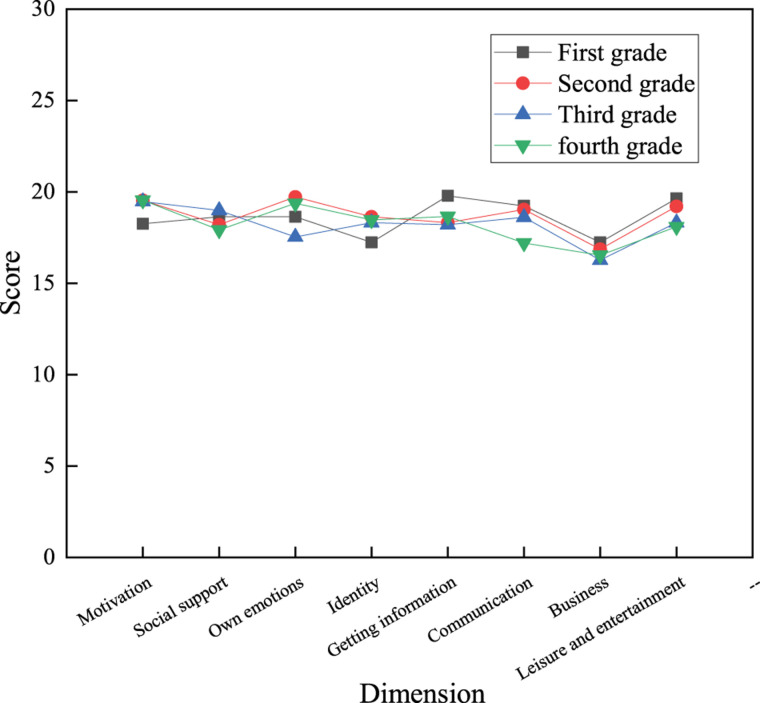
Influences of grades on the use of network buzzwords by college students.

According to Figure 10, in comparison, regarding behavioral factors, the communication scores of first-, second-, third-, and fourth-grade students are 19.24, 19.04, 18.62, and 17.21 points, respectively; and their scores in the leisure and entertainment dimension are 19.64, 19.21, 18.32, and 18.10 points, respectively, indicating that the scores of these two dimensions gradually decrease as the grade increases.

The above results suggest that college students of different genders, majors, and grades have different psychological and behavioral factors of using network buzzwords. Generally, boys score higher than girls because they spend much time on the internet; hence, they receive new information from the internet faster and more frequently than girls. As for different majors, students majoring in science and engineering score higher because the surveyed science and engineering students use the internet more frequently and think more actively. Hence, they are more likely to accept network buzzwords than those of other majors. Lower-grade students achieve higher scores in using network buzzwords because, as they just enter the school, they strongly demand to integrate into the new environment and new groups. Hence, they spend much energy on the sense of identity of the group. As an essential component of communication between students, network buzzwords are also easier to be accepted by the lower grade students. The higher-grade students have higher academic pressure, higher education pressure, and employment pressure, and they often have fewer opportunities to participate in group activities and online leisure and entertainment. Hence, they use network buzzwords less frequently.

## Discussion and Analysis

Situations and characteristics of Generation Z college students using network buzzwords are revealed by survey results. During daily communication, the primary psychological factor of college students using network buzzwords is identity. When college students enter an unfamiliar environment, using network buzzwords can shorten communication distance, making them recognized by the group more easily. Among the behavioral factors, the primary communication mode among college students is using network buzzwords. This result is also consistent with the characteristics of Generation Z. Network buzzwords significantly affect the daily lives of college students, which include learning, communication, and entrepreneurship (Chen, 2019).

Students of different genders, majors, and grades use network buzzwords differently. Overall, boys use network buzzwords more frequently than girls, because they spend more time and energy on the internet. Regarding students of different grades, those of lower grades use network buzzwords more frequently because they have just entered school and their willingness to integrate into the collective is pronounced. Students of higher grades undertake more substantial academic pressure and employment pressure; hence, they use more mature language in their daily lives. Using network buzzwords promotes personalization and emotionalization of information exchange, enabling people to gain a sense of belonging and identity in the virtual community, establish mutual trust invisibly, and finally, improve life satisfaction (Deng et al., 2021).

Science and engineering students use network buzzwords more frequently than those majoring in literature, history, and art. Professional courses for science and engineering students are somehow complex and tedious. Therefore, they are more willing to express their personalities, opinions, and thoughts in the virtual world. This is why science and engineering students pay more attention to the creation and development of language. In daily communication, they are more inclined to use network buzzwords.

Network buzzwords are also two-sided. On the one hand, they enable college students to better communicate with the outside world, obtain information, and enjoy the lively atmosphere, exerting positive influences. On the other hand, however, network buzzwords also include some terrible information, such as pornography, violence, and vulgarity. Families, colleges, universities, network media, and society should pay attention to network buzzwords, create a sound environment for college students to use network buzzwords, set positive examples and demonstrations, and strengthen awareness of language norms in college students. College students themselves should also improve their discrimination abilities and language cultivation, forming their unique cognitive system. Families should play an exemplary role, use standardized and civilized network buzzwords, and improve the civilized family environment. Colleges and universities should optimize the language environment in campus. Teachers should carefully identify the meanings and attributes of network buzzwords; those that are well-formed, active, and healthy can be used in classroom teaching activities. Government departments should strengthen network supervision, increase network law enforcement, and minimize the proliferation of bad ideas and network buzzwords that seriously violate social morality. Online media should strengthen self-audit, improve platform self-regulation, and create a sound online environment for netizens.

The previous research has only analyzed the theories and lacked actual survey data (Zheng et al., 2018). The surveys and interviews in this study make up for this research limitation. The research method combines theory and case investigation to analyze current situations of college students using network buzzwords based on field investigation data. Based on the background of the current era, the research results have practical application significances.

## Conclusion

The psychological mechanism of the use of network buzzwords by college students is explored from the perspective of educational psychology with targeted questionnaires and interviews based on psychological factors and behavioral factors. The main conclusions are as follows: first, college students are extremely familiar with network buzzwords. Meanwhile, among the psychological factors of the use of network buzzwords by college students, identity accounts for a large proportion, while social support accounts for a small proportion. In terms of behavioral factors, students get the highest score in the communication dimension, with an average score of 22.84 points. Second, communication is the most critical behavior mode for college students to use network buzzwords. Besides, the use of network buzzwords of college students is influenced by gender, major, and grade. Boys generally score higher than girls. The scores of science and engineering students are higher than those of other majors. Moreover, the scores of students in communication and entertainment activities gradually decrease with the increase in grades. The psychological and behavioral factors of the use of network buzzwords by college students are analyzed, involving students of different genders, majors, and grades. The research results have important significance and influence on the ideological education activities of civilized online behavior in colleges and universities. According to the above results, colleges, and universities can design targeted ideological education for different students. The research results have clearer purposiveness than previous studies and reveal the actual situation of the use of network buzzwords of college students more comprehensively. The deep exploration of the psychological mechanism of the use of network buzzwords of college students can provide a scientific and practical reference for the analysis and evaluation of the network behavior of college students and play an important role in the network psychology of college students. However, because of some objective constraints, there are fewer available samples for reference. Besides, the effect of network buzzwords on college students and ecological civilization construction has not been investigated for a sufficient time. Further detailed studies should be conducted to investigate the impact of network buzzwords on university education with a larger research scope and to evaluate the effect of the use of network buzzwords by generation Z college students in the era of “e-Learning 4.0.”

## Data Availability Statement

The raw data supporting the conclusions of this article will be made available by the authors, without undue reservation.

## Ethics Statement

The studies involving human participants were reviewed and approved by the Nanjing University Ethics Committee. The patients/participants provided their written informed consent to participate in this study. Written informed consent was obtained from the individual(s) for the publication of any potentially identifiable images or data included in this article.

## Author Contributions

The author confirms being the sole contributor of this work and has approved it for publication.

## Conflict of Interest

The author declares that the research was conducted in the absence of any commercial or financial relationships that could be construed as a potential conflict of interest.

## Publisher’s Note

All claims expressed in this article are solely those of the authors and do not necessarily represent those of their affiliated organizations, or those of the publisher, the editors and the reviewers. Any product that may be evaluated in this article, or claim that may be made by its manufacturer, is not guaranteed or endorsed by the publisher.
